# The Link Between *VDR* Gene rs7975232 Polymorphism and Benign Proliferative Breast Disease in Ukrainian Population

**DOI:** 10.1155/bmri/5536121

**Published:** 2026-02-23

**Authors:** Olha Obukhova, Mykola Kyrychenko, Ivan Lukavenko, Viktoriia Yu. Harbuzova

**Affiliations:** ^1^ Department of Physiology and Pathophysiology With Medical Biology Course, Medical Institute of the Sumy State University, Sumy, Ukraine, sumdu.edu.ua; ^2^ Department of Surgery, Traumatology, Orthopedics and Phthisiology, Medical Institute of the Sumy State University, Sumy, Ukraine, sumdu.edu.ua

**Keywords:** benign mammary dysplasia, benign proliferative breast disease (BPBD), gene polymorphism, *VDR*

## Abstract

**The Aim:**

We are aimed at examining the potential link between the *VDR* gene polymorphism rs7975232 and the occurrence of benign breast disease in the Ukrainian population.

**Materials and Methods:**

One hundred and six patients with BBD and 221 control subjects were enrolled in this case‐control study. PCR‐RFLP was used for *VDR* gene rs7975232 genotyping. SPSS software package (Version 25.0, IBM, USA) was used for data analysis.

**Results:**

There was a research relationship between the rs7975232 polymorphism and BPBD and other influencing factors as family history burden, predisposing factors, and blood estradiol levels, smoking, gynecological pathology, hormone intake, goiter, or mastodynia. In individuals younger than 40, heterozygotes have a higher BPBD risk compared with both homozygotes (*p* = 0.04), but this risk is not statistically significant after adjusting for BMI (*p* = 0.16). Conversely, for those aged 40 and above, heterozygotes have a lower BPBD risk compared with both homozygotes (*p* = 0.036), and this significant association remains after BMI adjustment (*p* = 0.012); additionally, recessive allele homozygotes have a higher BPBD risk compared wiht major allele carriers post‐BMI adjustment (*p* = 0.033).

**Conclusion:**

Obtained data suggested that rs7975232 polymorphism of the *VDR* gene can be a possible genetic marker for the development of benign proliferative breast disease in the Ukrainian population.

## 1. Introduction

Benign breast disease (BBD) is prevalent among women and significantly affects their quality of life. The frequency of BBD in the general population is 35%–50%, and among women of reproductive age, it is 67%–95%. Certain histological types of BBD also elevate the risk of developing breast cancer. As awareness about breast cancer grows, more women are presenting at hospitals with breast lumps, many of which are benign in nature. BBDs encompass a diverse array of conditions, ranging from developmental abnormalities and inflammatory lesions to epithelial and stromal proliferation, as well as neoplasms. The main etiological factor that triggers changes in the breast is considered to be an imbalance of estrogens. Under the influence of steroid hormones, proliferative activity of glandular epithelial cells occurs. [1–3].

One of the modern methods for diagnosing benign proliferative breast disease (BPBD), involves identifying genetic markers of this condition. Promising in this context are polymorphic variants of the vitamin D receptor (*VDR*) gene—a nuclear receptor that, along with estrogen, progesterone, and androgen receptors, is part of the nuclear family of transcription regulators for steroid hormones [4]. Vitamin D exerts pleiotropic effects on various tissues, including cancerous tumors. Its inhibition of breast cancer growth occurs through the activation of the *VDR* and classical nuclear signaling pathways. Presently, besides the traditional role of vitamin D in regulating calcium levels in the body, there are also acknowledged nontraditional effects: anti‐inflammatory, antiproliferative, and proapoptotic. Research has shown that *VDR* controls the expression of around 200 genes affecting cell differentiation, proliferation, and apoptosis. [5–7]. Several research studies have demonstrated that 1,25(OH) and similar compounds decelerate the proliferation of tumor cells, particularly during the G0/G1 phase of the cell cycle through apoptosis induction. Moreover, 1,25(OH) suppresses angiogenesis, cellular adhesion, and migration, consequently diminishing tumor cell proliferation. [8, 9]. Currently, over 25,000 polymorphisms of the *VDR* gene have been studied, and some of them, including rs7975232, are associated with the development of both benign and malignant tumors in various populations worldwide [10–13].

Regarding the Ukrainian population, the results concerning the impact of the rs7975232 polymorphism on the development of BPBD are contradictory and inconclusive. Information regarding its association with proliferative benign breast dysplasia is lacking. Therefore, we have initiated our own study aimed at investigating the role of the rs7975232 polymorphism of the *VDR* gene in the development of premenstrual dysphoric disorder in patients from the Sumy region of Ukraine. It is important to note that understanding the influence of *VDR* polymorphisms will contribute to a deeper understanding of the issue of benign tumor processes in the breast, which will facilitate the development of advanced methods for early diagnosis and prognosis of these diseases.

## 2. The Aim

The aim of the present work was to test the possible association between *VDR* gene rs7975232 polymorphic variant and the development of BPBD in female patients from the Sumy region of Ukraine.

## 3. Materials and Methods

### 3.1. Study Population

A total of 327 women residing in the Sumy region of Ukraine participated in this study, including 221 patients diagnosed with BPBD (the main group) and 106 women without BPBD (the control group). The median age of the BPBD group was 33.0 years (26.0–41.0), whereas the control group was slightly higher at 36.0 years (27.5–44.0). However, there was no statistically significant difference in age between the groups (*p* = 0.058). Patients were selected based on characteristics commonly considered indications for genetic testing for breast cancer [14], including multiple primary tumors in a single organ or in different organs, bilateral tumors in paired organs, multifocality within one organ, early tumor onset (before 21 years of age), and family history criteria such as one or more close relatives with the same type of tumor, two or more relatives with tumors of the same localization or related to familial cancer syndromes, rare cancer forms, or three or more relatives in two generations with tumors of the same localization. Exclusion criteria included nonproliferative breast changes, absence of signs of genetic predisposition to breast disease, and patient refusal to participate.

The protocol research was approved by the Bioethics Commission of the Educational and Scientific Medical Institute of Sumy State University (No. 3, 05.12.22), and complies with the Order of the Ministry of Health of Ukraine No. 690 dated September 23, 2009 (about the approving of conducting of medicines clinical trials procedure and expertise of clinical trials materials, and the model regulations on Ethics Committees: Order of the Ministry of Health of Ukraine No. 690. September 23, 2009 Ukrainian) and the Declaration of Helsinki of the World Medical Association on the ethical principles of conducting scientific medical research involving human subjects. All participants provided written informed consent for the processing of personal data, blood sampling, and participation in genetic analysis. Patients were examined on an outpatient basis in the office of a surgeon who operates in accordance with licensing conditions (License Ag No. 600519) and were operated on at the clinical bases of the department of surgery with a course in pediatric surgery and urology, including the Sumy Regional Clinical Oncology Center (Sumy, Ukraine). Morphological material was studied at the Pathomorphological Research Center of the Pathological Anatomy Department of Sumy State University, and molecular‐genetic studies were conducted at the Scientific Laboratory of Molecular‐Genetic Research of Sumy State University.

### 3.2. Genotyping

**Figure 1:**
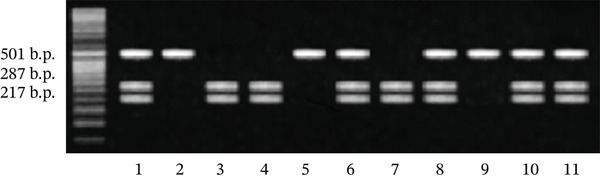
Results of *Apa*I restriction analysis of the *VDR* gene polymorphism: Lanes 3, 4, and 7 correspond to the a/a genotype; 1, 6, 8, 10, and 11 to the a/A genotype; and 2, 5, and 9 to the A/A genotype.

DNA from blood leukocytes was extracted with the help of the commercial GeneJET Whole Blood Genomic DNA Purification Mini Kit (Thermo Fisher Scientific, United States). The genotyping of *VDR* gene polymorphic variant rs7975232 C/A marker was performed by the polymerase chain reaction (PCR) method followed by restriction fragment length polymorphism (RFLP) analysis, with detection through agarose gel electrophoresis. The amplification of the gene region containing the polymorphism site rs7975232 was carried out using a pair of specific primers: forward (sense) – 5 ^′^‐CAGAGCATGGACAGGGAGCAA‐3 ^′^ and reverse (antisense) –5 ^′^‐CACTTCGAGCACAAGGGGCGTTAGC‐3 ^′^. The primers were synthesized by Metabion (Germany). For amplification, 50–100 ng of DNA was taken and added to a mixture containing 5 *μ*l of 5× PCR buffer, 1.5 mM magnesium sulfate, 250 *μ*M of the four dNTPs, 15 pM of each primer, and 0.75 U of Taq polymerase (Fermentas, Lithuania). The total volume was brought up to 25 *μ*l with deionized water. PCR was conducted in a GeneAmp PCR System 2700 thermocycler (Applied Biosystems, United States). The amplification program was as follows: denaturation at 94°C (50 s), primer annealing at 64.5°C (45 s), and elongation at 72°C (1 min), for a total of 33 cycles. Subsequently, 6 *μ*l of the amplification product was incubated at 37°C for 20 h with 5 U of *Apa*I restriction enzyme in buffer B with the following composition: 10‐mM Tris‐HCl (pH 7.5), 10‐mM magnesium chloride, and 0.1‐mg/mL albumin. If there was a guanine at position 59979 of the *VDR* gene, the amplicon, which consisted of 501 base pairs, was cleaved by *Apa*I into two fragments—284 and 217 base pairs. If guanine was replaced with thymine, the restriction site for *Apa*I was lost, resulting in a single fragment of 501 base pairs (Figure 1).

The amplificates of the studied fragment of the *VDR* gene after restriction were separated in a 2.5% agarose gel containing ethidium bromide. Horizontal electrophoresis (0.1A; 140 V) was performed for 40 min. Visualization of DNA after electrophoresis was carried out using a transilluminator “Biocom”.

### 3.3. Data Analysis

Statistical analysis of the data was conducted with the SPSS software package (Statistical Package for the Social Sciences, Version 25.0, IBM, United States). To assess whether continuous data followed a normal distribution, the Kolmogorov–Smirnov test was applied. All continuous variables are reported as the mean and standard deviation (M ± SD). The Pearson *χ* ^2^ test was used to evaluate whether the frequency distribution of the rs7975232 genotypes *VDR* gene aligned with the Hardy–Weinberg equilibrium. A similar comparative analysis was also conducted to examine genotype distribution and other categorical variables across the study groups, using the *χ* ^2^ test. To compare the M values between two groups, the Student′s t‐test for independent samples was applied. When comparing the M values across three different rs7975232 genotypes, the ANOVA method was used, followed by the Bonferroni post hoc test for more detailed analysis. To evaluate the risk of BPBD complications in relation to the specific rs7975232 genotype, binary logistic regression was utilized. Multivariable logistic regression was employed to account for potential confounding factors such as sex, age, and body mass index. A p value below 0.05 was considered statistically significant across all tests.

## 4. Results

The distribution of *VDR* rs7975232 genotypes are as follows: in the control group a − allele frequency = 0.547 and A − allele = 0.453, in the BPBD group the frequency a − allele = 0.545 and A − allele = 0.455. The allele frequency distribution in the comparison groups aligns with the Hardy–Weinberg distribution (p = 0.963, *χ* ^2^ = 0.002).

Table 1 presents the outcomes of genotyping for the *VDR* gene rs7975232 in both cohorts.

**Table 1:** The frequency of *VDR* rs7975232 genotypes in the study groups.

Group	*n*	Genotype			*p* (*χ* ^2^)
		a/a (%)	a/А (%)	A/A (%)	
**Benign proliferative breast disease (case group)**					
Control	106	34 (32.1)	48 (45.3)	24 (22.6)	0.868 (0.282)
Case	221	67 (30.3)	107 (48.4)	47 (21.3)	

*Note:* n—number of subjects. *χ* ^2^test was used for data comparison.

No statistically significant differences were found in the distribution of genotypes and alleles between the comparison groups (p > 0.05).

Table 2 presents the results of the analysis of the association between genotypes for the rs7975232 polymorphism of the *VDR* gene and BPBD, conducted using binary and multivariable logistic regression within the framework of four inheritance models.

**Table 2:** Analysis of *VDR* rs7975232 genotypic association with the risk of BPBD development.

Model	*p*_*o* *b* *s*_	OR_obs_ (95% CI)	*p*_*a* *d* *j*_	OR_*a* *d* *j*_ (95% CI)
**Benign proliferative breast disease (case group)**				
Dominant	0.747	1.085 (0.659–1.787)	0.918	1.028 (0.605–1.748)
Recessive	0.778	0.923 (0.529–1.612)	0.584	1.184 (0.647–2.164)
Over dominant	0.595	1.134 (0.713–1.805)	0.715	0.911 (0.553–1.502)
Additive^a^	0.652	1.131 (0.663–1.932)	0.802	0.928 (0.52–1.658)
	0.985	0.994 (0.523–1.888)	0.753	1.116 (0.562–2.216)

*Note:* The “obs” index refers to the results of logistic regression without adjustments; the “adj” index indicates the results of logistic regression after adjusting for age and body mass index.

^a^The top row shows the results of comparing a/A versus a/a, and the bottom row shows A/A versus a/a.

In all inheritance models, both before and after adjusting for covariates (age and body mass index), there was no association found between the rs7975232 polymorphism and the risk of BPBD development (*p* > 0.05).

The subsequent stage of the analysis involved examining the risk of BPBD development in women across various age groups with diverse genotypes for the rs7975232 polymorphism of the *VDR* gene (Table 3).

**Table 3:** Analysis of the association between the polymorphism rs7975232 and the development of BPBD depending on the age of the patients.

Model	*p*_*o* *b* *s*_	OR_obs_(95%CI)	*p*_*a* *d* *j*_	OR_*a* *d* *j*_(95%CI)
**Less than 40 years old**				
Dominant	0.524	1.227 (0.653–2.305)	0.665	1.165 (0.585–2.32)
Recessive	0.066	0.522 (0.261–1.044)	0.211	0.611 (0.283–1.321)
Over dominant	0.040	1.858 (1.029–3.357)	0.160	1.588 (0.833–3.025)
Additive^a^	0.181	1.596 (0.805–3.165)	0.352	1.421 (0.678–2.975)
	0.372	0.694 (0.312–1.547)	0.537	0.759 (0.316–1.824)
**40 years and older**				
Dominant	0.694	0.847 (0.372–1.931)	0.565	0.779 (0.333–1.825)
Recessive	0.057	2.652 (0.971–7.245)	0.033	3.119 (1.097–8.868)
Overdominant	0.036	0.424 (0.19–0.943)	0.012	0.342 (0.147–0.794)
Additive^a^	0.185	0.545 (0.222–1.227)	0.095	0.446 (0.173–1.152)
	0.261	1.907 (0.619–5.869)	0.215	2.125 (0.645–7.001)

*Note:* The “obs” index refers to the results of logistic regression without adjustments; the “adj” index indicates the results of logistic regression after adjusting for body mass index.

^a^The top row shows the results of comparing a/A versus a/a., and the bottom row shows A/A versus a/a.

In the group with less than 40 years old according to the overdominant model, heterozygotes have a higher risk of developing BPBD than both homozygotes (*p* = 0.04). However, after adjusting for BMI, the association was no longer statistically significant (*p* = 0.16). In the group of patients aged 40 and older, according to the overdominant model, heterozygotes have a lower risk of developing BPBD compared with both homozygotes (*p* = 0.036). The statistically significant association remained after adjusting for BMI (*p* = 0.012). According to the recessive inheritance model, homozygotes for the recessive allele have a higher risk of developing BPBD compared with carriers of the major allele after adjusting for BMI (*p* = 0.033) (Table 3).

We also studied the association of the rs7975232 polymorphism in the *VDR* gene with disease duration, age at menarche onset, cycle length, and duration of menses in patients with BPBD. (Table 4).

**Table 4:** Comparison of disease duration, age at menarche onset, cycle length, and duration of menses in patients with BPBD depending on genotype according to rs7975232 polymorphism.

	Genotype			
	а/а (*n* = 67)	а/А (*n* = 107)	А/А (*n* = 47)	*p*
Disease duration, years	3.8 ± 2.9	3.2 ± 2.9	3.1 ± 2.8	0.438
Age at menarche onset, years	13.1 ± 1.3	13.3 ± 1.5	13.4 ± 1.5	0.572
Cycle length, days^a^	25.9 ± 6.9	27.8 ± 3.9	24.8 ± 8.8	0.017
Duration of menses, days	5.1 ± 3.2	4.8 ± 1.5	4.6 ± 1.9	0.352

^a^After applying the Bonferroni correction, statistically significant differences were found between the genotypes a/A and A/A (*p* = 0.024).

It was found that among carriers of different genotypes for the rs7975232 polymorphism, patients with BPBD have differences in the duration of menses. Specifically, A/A homozygotes have a significantly shorter duration of menses compared with a/A heterozygotes (A/A = 24.8 ± 8.8, a/A = 27.8 ± 3.9; p = 0.024) (Table 4).

We also investigated the distribution of genotypes for the rs7975232 polymorphism of the *VDR* gene in patients with BPBD, considering other influencing factors, namely, severity of anamnesis, predisposing factors, smoking, gynecological pathology, hormone intake, presence of goiter, mastodynia, and estradiol levels (Table 5).

**Table 5:** Comparison considering other factors in patients with BPBD depending on the genotype of the rs7975232 polymorphism.

	Genotype			
	а/а, *n* (%)	а/А, *n*(%)	А/А, *n*(%)	*p* (*χ* ^2^)
	**Severity of anamnesis**			
Not burdened	45 (67.2)	86 (80.4)	26 (55.3)	0,005 (10.667)
Burdened	22 (32.8)	21 (19.6)	21 (44.7)	
	**Predisposing factors**			
Present	24 (36.9)	34 (34.7)	27 (61.4)	0.008 (9.596)
Absent	41 (63.1)	64 (65.3)	17 (38.6)	
	**Smoking**			
Nonsmoker	47 (70.1)	78 (72.9)	35 (74.5)	0.868 (0.284)
Smoker	20 (29.9)	29 (27.1)	12 (25.5)	
	**Gynecological pathology**			
Absent	32 (47.8)	50 (46.7)	20 (42.6)	0.848 (0.329)
Present	35 (52.2)	57 (53.3)	27 (57.4)	
	**Hormonal medications**			
Not taking	54 (80.6)	83 (79)	32 (69.6)	0.337 (2.175)
Taking	13 (19.4)	22 (21)	14 (30.4)	
	**Goiter**			
Absent	54 (80.6)	95 (88.8)	40 (85.1)	0.326 (2.239)
Present	13 (19.4)	12 (11.2)	7 (14.9)	
	**Mastodynia**			
Absent	26 (39.4)	50 (46.7)	22 (46.8)	0.603 (1.013)
Present	40 (60.6)	57 (53.3)	25 (53.2)	
	**Estradiol levels**			
Not increased	51 (79.7)	96 (93.2)	44 (97.8)	0.003 (11.859)
Increased	13 (20.3)	7 (6.8)	1 (2.2)	

*Note:* Table 5 presents the analysis of additional clinical and laboratory factors in the cohort of 221 BPBD patients depending on the genotype of the rs7975232 polymorphism. The effective number of observations differed between variables due to missing values: Data on predisposing factors were available for 207 patients, current hormonal medication intake for 218 patients, mastodynia status for 220 patients, and estradiol levels for 212 patients. Percentages were calculated based on the actual number of patients with complete information for each specific parameter.

In the study of the distribution of genotype frequencies of the rs7975232 polymorphism in the *VDR* gene by family history burden in patients with BPBD, statistically significant differences were found. The frequency of patients with a family history burden in the A/A‐homozygote group was higher than in the a/A heterozygote and a/a homozygote groups (Table 5).

Statistically significant differences were also found in the presence of predisposing factors among genotypes of the *VDR* gene polymorphism. In patients with the A/A genotype, predisposing factors were more frequently present than in individuals with genotypes a/a and a/A (Table 5).

Statistically significant differences were found in blood estradiol levels between genotypes: Elevated estradiol levels were more frequently observed in carriers of the a/a genotype compared with a/A and A/A genotypes (Table 5).

When considering other factors such as smoking, presence of gynecological pathology, hormone intake, presence of goiter, and mastodynia in patients with BPBD, no statistically significant differences were found based on the genotype of the rs7975232 polymorphism (Table 5).

## 5. Discussion

The endocrine function of vitamin D is crucial for multiple biological processes, including regulation of bone metabolism, modulation of immune responses, and control of cell growth and differentiation [15]. Extensive genetic variability has been identified within the *VDR*, with more than 25,000 reported variants, although most remain functionally unexplored.

The *VDR* gene is located on Chromosome 12 (12q13.11) and consists of 11 exons and five promoter regions [16]. SNP rs7975232 involves a substitution of guanine with thymine in the eighth intron at position 59979. Although intronic polymorphisms do not alter the coding sequence, their proximity to regulatory regions allows them to serve as markers of functional interactions between other SNPs and pathological processes [17–19].

Polymorphisms in the *VDR* gene have been studied across different ethnic populations, demonstrating significant racial and ethnic differences in allele frequencies [18]. These disparities are attributed to evolutionary and population‐genetic processes. Consequently, individual polymorphisms may be associated with variable disease prevalence among ethnic groups.

Our study investigated the association of the rs7975232 polymorphism with BPBD in female patients from the Sumy region of Ukraine. This research addresses a notable gap, as no previous data exist on the role of this variant in Ukrainian women and only limited information is available regarding *VDR* polymorphisms in tumor‐related conditions within the Ukrainian population.

The analysis showed age‐dependent associations under the overdominant inheritance model. In patients younger than 40 years, heterozygotes had a higher BPBD risk compared with homozygotes (*p* = 0.04), but this association lost significance after adjustment for BMI (*p* = 0.16). In women aged 40 and older, heterozygotes demonstrated a lower BPBD risk (*p* = 0.036), which remained significant after BMI adjustment (*p* = 0.012). The recessive model also indicated increased BPBD risk in homozygotes for the recessive allele following BMI correction (*p* = 0.033). These findings suggest that genotype influences BPBD susceptibility, with age and BMI acting as important modifying factors.

Statistically significant differences were observed in genotype distribution, with a higher prevalence of family history burden in A/A homozygotes. Elevated estradiol levels and presence of predisposing factors were more common in certain genotypes, whereas no significant associations were detected with smoking, gynecological pathology, hormone intake, goiter, or mastodynia.

Previous international studies support the involvement of VDR polymorphisms and vitamin D signaling in breast cancer. Variants *Apa*I and *Taq*I have been linked to increased breast cancer risk [20], and deregulation of *VDR* and vitamin D metabolic enzymes (CYP27B1 and CYP24A1) has been shown to promote tumor progression [21]. In Ukraine, *VDR* gene polymorphisms have already been recognized as risk markers for several nontumor diseases, including generalized parodontitis [22] and ischemic atherothrombotic stroke [23], and are being evaluated as modifiers of hypertension risk in stroke patients [24]. The role of polymorphisms of hormone–receptor genes in breast pathology has also been investigated, particularly the *VDR* variants *Fok*I, *Bsm*I, *Apa*I and *Taq*I [24], as well as the *Pvu*II polymorphism of the estradiol receptor alpha gene for diagnosis of proliferative forms of benign breast dysplasia [25].

Thus, the rs7975232 polymorphism appears to be a meaningful genetic marker in BPBD development among Ukrainian women. Investigation of additional VDR polymorphisms will allow identification of haplotypes associated with both BPBD and breast cancer [18].

## 6. Conclusions

This is the first case‐control study to analyze the relationship between *VDR* genetic polymorphism rs7975232 and BPBD in the Ukrainian population. It was found that in individuals under 40, heterozygotes have a higher risk of developing BPBD compared with both homozygotes (*p* = 0.04), but this association becomes insignificant after adjusting for BMI (*p* = 0.16). In those aged 40 and older, heterozygotes have a lower risk of developing BPBD compared with both homozygotes (*p* = 0.036), and this association remains significant even after BMI adjustment (*p* = 0.012). Additionally, homozygotes for the recessive allele have a higher risk of developing BPBD compared with carriers of the major allele after BMI adjustment (*p* = 0.033).

Thus, age and genetic model play crucial roles in the risk of developing BPBD, with BMI adjustment affecting statistical significance differently across age groups and genetic models.

Carriers of different genotypes for the rs7975232 polymorphism in the *VDR* gene show significant differences in the duration of menses among BPBD patients, with A/A homozygotes having a shorter duration compared with a/A heterozygotes (A/A = 24.8 ± 8.8, a/A = 27.8 ± 3.9; *p* = 0.024). Also, in patients with BPBD, the rs7975232 polymorphism of the *VDR* gene shows significant genotype‐based differences in family history burden, presence of predisposing factors, and blood estradiol levels, but not in smoking, gynecological pathology, hormone intake, goiter, or mastodynia.

Expanding the scope of research to include larger, multiethnic cohorts would provide valuable insights into population‐specific genetic variations and their implications for BPBD and other diseases. This approach could help distinguish universal patterns from region‐specific genetic predispositions, offering a deeper understanding of evolutionary factors shaping *VDR* polymorphism distributions. Additionally, longitudinal studies could shed light on the dynamic interplay between *VDR* polymorphisms, environmental exposures, and age‐related changes in breast tissue biology.

## Author Contributions

**Olha Obukhova:** conceptualization, methodology, carried out molecular genetic studies, and writing—original draft. **Mykola Kyrychenko:** carried out molecular genetic studies and formal analysis. **Ivan Lukavenko:** data collection, analysis, and writing—review and editing. **Viktoriia Yu. Harbuzova:** conceptualization, supervision, project administration, and review and editing.

## Funding

This study was supported by the Ministry of Education and Science of Ukraine (10.13039/501100007684) (No. 0123U101850).

## Disclosure

The present study is part of the project supported by Ministry of Education and Science of Ukraine (No. 0123U101850).

## Conflicts of Interest

The authors declare no conflicts of interest.

## Data Availability

The data that support the findings of this study are available on Google Drive at the following link: https://docs.google.com/document/d/16Zo46zYHgPpAeGndHQM-7mEJI3FDiOBI/edit?usp=drive_link%26ouid=118170371067094484183%26rtpof=true%26sd=true. The data will be available upon reasonable request.
